# Quantification of Venetoclax for Therapeutic Drug Monitoring in Chinese Acute Myeloid Leukemia Patients by a Validated UPLC-MS/MS Method

**DOI:** 10.3390/molecules27051607

**Published:** 2022-02-28

**Authors:** Xi Yang, Chen Mei, Xiaoying He, Lingjuan He, Xiaoyang Lu, Hongyan Tong, Yan Lou

**Affiliations:** 1 Key Laboratory for Drug Evaluation and Clinical Research of Zhejiang Province, Department of Clinical Phamacy, The First Affiliated Hospital, College of Medicine, Zhejiang University, 79 QingChun Road, Hangzhou 310000, China; 1516100@zju.edu.cn (X.Y.); hxy17858505013@163.com (X.H.); hlingj19@163.com (L.H.); luxiaoyang@zju.edu.cn (X.L.); 2 Department of Hematology, The First Affiliated Hospital, College of Medicine, Zhejiang University, 79 QingChun Road, Hangzhou 310000, China; meichenblood@yeah.net

**Keywords:** venetoclax, UPLC-MS/MS, therapeutic drug monitoring

## Abstract

Venetoclax has emerged as a breakthrough for treatment of leukemia with a wide interindividual variability in pharmacokinetics. Herein, a rapid, sensitive, and reliable UPLC-MS/MS method for quantification of venetoclax in plasma was developed and validated. The method was operated in the multiple-reaction monitoring (MRM) mode to detect venetoclax at m/z transition 868.5 > 321.0 and IS at 875.5 > 321.0, respectively. Protein precipitation prior to injection into the LC-MS/MS and the analyte was separated on an ACQUITY UPLC BEH C18 column by gradient elution with acetonitrile and 0.1% formic acid in water. Linear calibration curves were obtained in the range of 25–8000 ng/mL. The specificity, recovery, matrix effect, and stability also met the acceptance criteria of FDA guidance. The method was successfully applied to analyze plasma in acute myeloid leukemia (AML) patients. The peak plasma concentration (C_max_) of venetoclax in Chinese AML patient was 2966.0 ± 1595.0 ng/mL while the trough concentration (C_min_) was 1018.0 ± 729.4 ng/mL. Additionally, C_max_ and C_min_ showed a positive correlation with AST levels. Furthermore, C_max_ was significantly higher in the older patients. The present method can be applied for TDM of venetoclax in treatment of hematological cancers.

## 1. Introduction

Hematological malignancy constitutes serious, high-relapse, and hardly curable malignant tumors. It is characterized by an unchecked proliferation and blocked differentiation of abnormal hematopoiesis stem and progenitor cells, which is accompanied by an obstructed apoptosis [1]. The conventional chemotherapy is unsuitable for treatment in the elderly or patients who cannot tolerate it and chemotherapy resistance is a major cause of treatment failure [2,3]. In recent years, targeted therapies in certain aspects of leukemia cell processes such as proliferation and apoptosis brought the improvement of new strategies to treat hematological malignancy [2]. The B cell leukemia/lymphoma-2 (BCL-2), an intrinsic anti-apoptotic protein, is aberrantly overexpressed in leukemia stem cells. It plays a vital role in obstruction of leukemia cell apoptosis and drug resistance [4,5,6].

Venetoclax is an excellent orally selective inhibitor via competing for the BH3 binding site on BCL-2, thereby changing the mitochondrial outer membrane permeability, activating the caspase, and then restoring the malignant cells’ apoptosis pathway [7,8]. In April 2016, the FDA granted accelerated approval to venetoclax as monotherapy for treatment in patients diagnosed with CLL /SLL with chromosome 17p deletion who have received at least one prior therapy [9]. Subsequently, the FDA expanded venetoclax approval in combination with hypomethylating agent drugs (HMAs) for AML patients who are at least 75 years old or unsuitable for intensive induction chemotherapy [10]. Meanwhile, Further trials involving the use of venetoclax in combination therapies are also still being conducted in other malignancies [11].

Due to its high selectivity for the BCL-2 protein, venetoclax shows superior efficacy and better safety profiles compared with other drugs [12,13]. However, the continued use of venetoclax at high doses can result in life-threatening tumor lysis syndrome (TLS, 12%) and the most frequent adverse events including neutropenia (45%), diarrhea (35%), nausea (33%), anemia (29%), and thrombocytopenia (22%) [14]. Furthermore, venetoclax has a high inter-patient’ variability with nonlinearity in relative bioavailability [15,16]. According to its pharmacokinetics, venetoclax reaches peak plasma concentration 5–8 h after an oral administration and has a high plasma protein bound rate (>99%) with a large apparent volume of distribution [9]. Venetoclax is predominantly metabolized by CYP3A4, also a substrate for OATP1B3, P-glycoprotein (P-gp), and breast cancer-resistance protein transporters (BCRP) [17]. Reports showed that the mean C_max_ of venetoclax was 94% higher in Chinese subjects compared with non-Asian subjects receiving the same dose [18].

Considering the wide interindividual variability in pharmacokinetics and therapeutic response of patients towards anticancer drugs, it is not possible to predict whether an individual patient will reach an adequate plasma exposure using a standard fixed dose of the drug. Therapeutic drug monitoring (TDM) is the measurement of specific drugs’ stable concentration in a patient’s bloodstream, thereby optimizing individual dosage regimens [19]. It is commonly and successfully used to ensure appropriate drug exposure and to limit dose-related toxicities [20]. TDM is cost-effective, and it could be beneficial to personalize a dose to fit the specific needs of cancer patients [21,22,23]. At present, TDM results concerning venetoclax have few precedents in scientific literature, and there are only a few studies on the quantitation of venetoclax in biological fluids. These methods include liquid scintillation counting (LSC), high-performance liquid chromatography (HPLC), and liquid chromatography tandem mass spectrometry (LC-MS/MS) [24,25]. However, the above methods often require multiple sample preparation steps such as liquid-liquid extraction (LLE), solid phase extraction (SPE), and a long-running program. Choo et al. mentioned a rapid LC-MS/MS method for quantification of venetoclax in dogs’ plasma within 1.6 min [26], but did not present the method validation; thus, the reproducibility of the method, perhaps, is uncertain. Reddy et al. developed a single and accurate LC-MS/MS method to detect venetoclax in rats’ plasma, over the range of 5–500 ng/mL under a time-consuming sample dilution [27]; however, it is unsuitable for TDM to capture inter-intra individual variability in case of overdose. Thus, it is necessary to develop a more sensitive and efficient method to detect venetoclax for high-throughput TDM analysis in humans.

The purpose of the work presented herein was to describe the development and validation of a convenient method for the rapid and reliable quantitation of venetoclax in human plasma. Additionally, the validated method was applied to the therapeutic drug monitoring of Chinese AML patients who were undergoing venetoclax therapy, and whether the peak and trough concentrations of venetoclax are related to the clinical features was explored, which resulted in the large interindividual variation of venetoclax exposure.

## 2. Results and Discussion

### 2.1. Method Development and Optimization

The optimizations of mass spectrum conditions for venetoclax and internal standard(IS) were acquired by continuous infusion of each analyte under the concentration of 500 ng/mL dissolved in ACN/water (50:50, *v*/*v*), via the mass spectrometer fluidic pump at a flow rate of 5 μL/min. Considering of the PKa value, the positive electrospray ionization (ESI^+^) mode was selected in our method with the two most abundant fragments of m/z 868.5 → 321.0, 868.5 → 636.3 for venetoclax, and *m*/*z* 875.5 → 321.0, 875.5 → 636.3 for IS; quantification and confirmation ions were monitored for each analyte. Subsequently, cone voltage and collision energy for those transitions were optimized to achieve sufficient MRM signal (Table 1).

The composition of the mobile phase directly affects the separation and ionization. Due to the pKa and logP values, the mobile phase comprised ACN and water containing 0.1% formic acid was chosen to improve chromatographic performance in our method. The total procedure, including washing and re-equilibrating steps, took 4 min, shorter than the time spent in previously published method. Although Choo et al. provided a faster separation method in only 1.6 min [26], the detail of gradient elution was not presented, and whether it contained the washing and equilibrating procedure is unknown. Without a washing procedure, the remaining salt or other plasma components may affect column performance and MS ionization. In addition, a limitation of the work of Choo et al. is lacking the comprehensive method validation. Furthermore, the composition of the mobile phase in our assay is more convenient than the method published by Choo et al. and Reddy et al., which added 2 mM ammonium acetate in water and acetonitrile with 0.1% formic acid [26,27]. Some other methods have been reported with a time-consuming and complicated SPE, and LLE for sample pretreatment [24,25]. However, the sample preparation strategy selected in our experiment was ACN protein precipitation with only single-step extraction, evaluated by matrix effect for three concentrations, which is a fast, cost-effective, and manageable procedure for routine measurements in clinical settings. In addition, the quantitative methods created by Choo et al. and Reddy et al. were used in determination of dog or rat plasma, rather than human plasma analysis [26,27]. Here, we provided the summary of analytical methods for quantification of venetoclax (Table S1, Supplementary Materials).

In our study, we described an advantageous analysis to determine venetoclax in human plasma over the published multiple-analyte method, which is characterized by a more laborious sample preparation procedure (e.g., liquid-liquid extraction) and a complex mobile phase (e.g., ammonium aqueous solution or ACN–MeOH, 50/50 *v*/*v*); meanwhile, our method seems more suitable for clinical high-throughput TDM analysis with a good sensitivity in a short time.

### 2.2. Method Validation

#### 2.2.1. Linearity, Selectivity and Specificity

**Figure 1:**
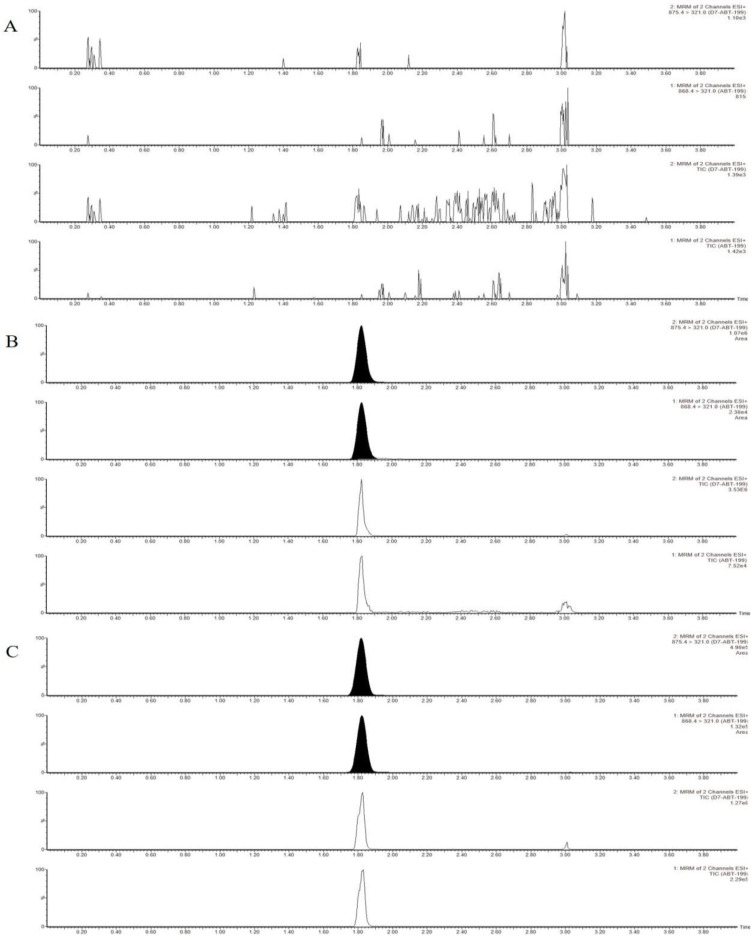
Representative UPLC-MS/MS chromatograms of venetoclax and IS (**A**)—blank plasma; (**B**)—blank plasma-spiking venetoclax and IS; (**C**)—plasma sample 0.5 h before continuous oral administration of venetoclax at steady state).

Typical MRM chromatograms of blank plasma, blank matrix spiked with IS, and venetoclax at a lower limit of quantification (LLOQ), as well as a sample from an AML patient during venetoclax treatment are presented in Figure 1. The retention time of venetoclax and IS were 1.8 and 1.8 min, respectively. No significant interfering peaks were observed around the retention time of venetoclax and IS, illustrating that the method was specific for the analyte determination.

**Figure 2:**
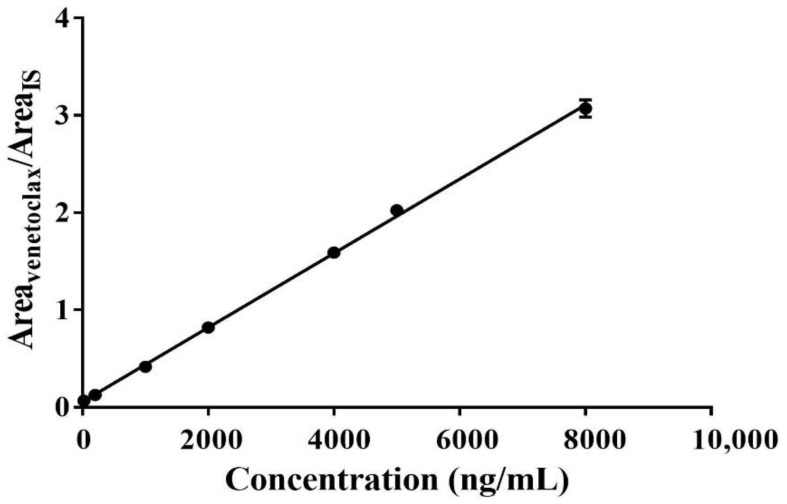
The linear relationship between the analyte concentration and the peak area ratios (*n* = 3).

The calibration curve of venetoclax was validated at seven points over the concentration range of 25–8000 ng/mL covering the plasma therapeutic range with a coefficient of correlation (r^2^ ≥ 0.997, Figure 2), following the regression equation (y = 0.0003816x + 0.05653). Each concentration was analyzed in three replicates and the precision for each calibrator with RSD ranged from 0.38% to 5.35%. According to several literatures, the plasma concentration of venetoclax varied from 30 to 4000 ng/mL in human (including healthy volunteers, chronic lymphocytic leukemia patients, and small lymphocytic lymphoma patients) across the dose levels (100–600 mg) [28,29,30,31], indicating that the calibration curve is capable of measuring the concentration of venetoclax in human plasma by encompassing both peak and trough concentrations.

#### 2.2.2. Precision and Accuracy

Results concerning precision and accuracy over low-concentration quality control (LQC), middle-concentration quality control (MQC), high-concentration quality control (HQC), and lower limit of quantification (LLOQ) samples are presented in Table 2. The intra-day and inter-day precision (relative standard deviation (%RSD)) for venetoclax ranged from 1.99% to 6.18% and 4.13% to 7.30%. Furthermore, the intra- and inter-day accuracy (relative error (%RE)) varied from −6.51%to 0.95% and −4.81% to 0.49%, respectively. Therefore, the results of precision and accuracy met the acceptable criteria of FDA guidelines.

#### 2.2.3. Recovery and Matrix Effect

Results of extraction recovery and matrix effect at three level of QCs concentrations are depicted in Table 3. The extraction recovery of venetoclax in human plasma was nearly 100% with an RSD ≤ 5.37%, while the extraction recovery of IS was 104.41 ± 2.76%. Matrix effects of the analyte was in the range of 84.5–95.2% with RSD ≤ 5.33%, and the IS matrix effect was 90.19% ± 2.34%. This demonstrated that the developed method had a highly efficient extraction and the matrix effect was concentration-independent.

#### 2.2.4. Carry-Over

Carryover was evaluated by the detection of blank sample following upper limit of quantification (ULOQ) samples, and there was no response at the corresponding retention times, indicating no carryover occurred.

#### 2.2.5. Stability

The results of stability at two QC concentration levels in plasma under a variety of conditions are summarized in Table 4. The accuracy (%RE) and precision (%RSD) were in the range of −8.66–0.76% and 1.0–4.6%, respectively. The results demonstrated a good stability of venetoclax throughout the experiment.

### 2.3. Patient Characteristics and Venetoclax Concentrations

The analysis included 109 venetoclax plasma concentrations from 62 AML patients. Their demographic and clinical characteristics are displayed in Table 5. Patients’ median age was 62 years (range, 18–90), and 36/62 (58.1%) were male.

**Figure 3:**
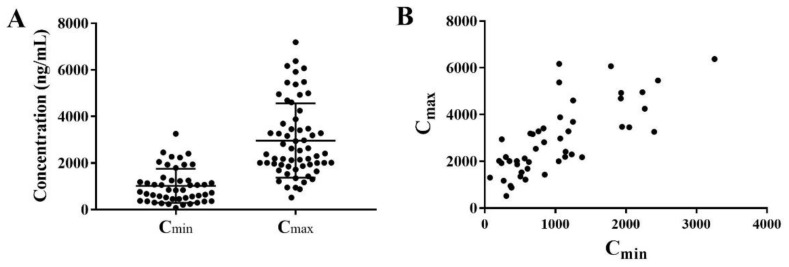
Distribution of venetoclax trough concentrations and peak concentrations in Chinese AML patients (**A**)—summary of venetoclax concentrations, (**B**)—paired of C_min_ and C_max_).

The developed UPLC–MS/MS method was applied to measure venetoclax concentrations in plasma. The peak plasma concentrations (C_max_) of venetoclax in Chinese AML patient were 2966.0 ± 1595.0 ng/mL, while the trough concentrations (C_min_) were 1018.0 ± 729.4 ng/mL (Figure 3). Both C_max_ and C_min_ exhibited a large interindividual variability, and this observation is consistent with previous studies.

### 2.4. Relationship between Covariates and Venetoclax Concentrations

**Figure 4:**
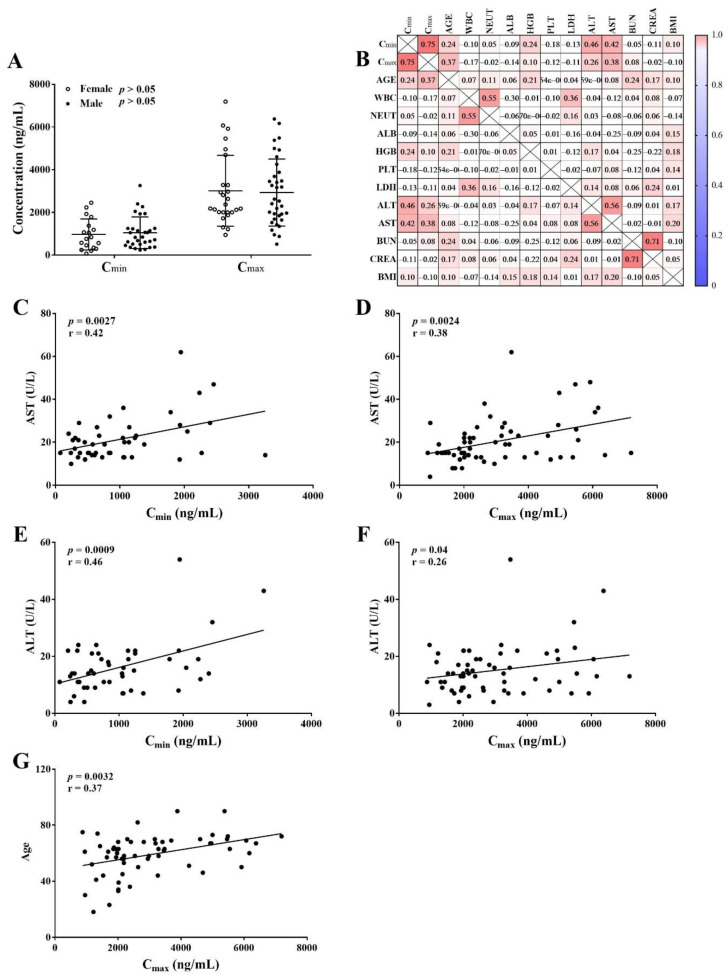
Variables associated with the venetoclax concentrations. The venetoclax concentrations in the female cohort were similar to the male cohort (**A**). The correlation heatmap between venetoclax concentrations and clinical features (**B**). The AST concentration and the venetoclax concentration were positively associated (**C**,**D**); The ALT showed a relevant effect on C_min_ rather than C_max_ (**E**,**F**); The age and the C_max_ of venetoclax were positively associated (**G**). WBC, white blood cell count; NEUT, neutrophil; HGB, hemoglobin; ALB, albumin; PLT, platelet; LDH, lactate dehydrogenase; ALT, alanine aminotransferase; AST, aspartate transaminase; BUN, blood urea; CREA, creatinine.

The C_min_ showed no difference between the female cohort and male cohort. Similarly, no correlation was observed between sex and C_max_ (Figure 4A), in line with the previous study performed by Deng et al. [32]. In the literature, venetoclax’s volume of distribution is 30% lower in females than in males, and this difference in apparent volume is not considered clinically relevant with C_max_ [33]. Correlation heatmap displayed the relationship between concentrations and the characteristics (Figure 4B). Red color indicates a positive correlation and blue color indicates a negative correlation. In addition, a significant correlation was observed between aspartate transaminase (AST) levels and venetoclax concentrations (C_min_ and C_max_), and AST showed a clinically relevant effect on C_min_ and C_max_ (*p* < 0.01, r > 0.3, Figure 4C–D). Moreover, alanine aminotransferase (ALT) showed a relevant effect on C_min_ (*p* < 0.01, r > 0.3, Figure 4E), but it had no clinically relevant effect on C_max_ (*p* > 0.01, r < 0.3, Figure 4E). However, Deng et al. did not find any effect on clearance with AST or ALT levels and estimated venetoclax in linear elimination [32]. Apart from the individual differences and insufficient sample size in our experiment, the divergence may due to the effects of ethnicity and type of leukemia on pharmacokinetics; only 1.19% of patients enrolled were Asian (1.19%) with CLL in Deng et al. [32], while our experiment was carried out in Chinese AML patients. Besides, there was no change of AST and ALT level after treatment. The comparison of AST and ALT levels before and after treatment is displayed in the Supplementary Materials (Figure S1). According to its pharmacokinetic process, venetoclax is predominantly eliminated by the liver. In view of USA National Cancer Institute (NCI) Organ Dysfunction Working Group for Hepatic Dysfunction, AST levels and ALT levels are used as indicators for evaluating liver function [34]. At the same time, it is noted that venetoclax dosage adjustment is required in subjects with severe hepatic impairment in another study, which recommended a 50% dose reduction of venetoclax [35]. It is important to monitor the concentration in hepatic impairment patients during venetoclax therapy.

Meanwhile, a markedly raised venetoclax peak concentration was associated with the growth of age (*p* < 0.01, Figure 4E), partially as a result of the decreased activity of intestine-CYP3A4 and P-gp in elderly patients. As apparent volume of distribution of venetoclax is very large (241 L) [9], a variation in albumin will have a rare impact on the variation in volume of distribution in spite of the large protein-bound rate. Consequently, a negligible influence on C_max_ and C_min_ by albumin was observed (*p* > 0.05; r = −0.09, −0.14).

Several factors may influence the systemic exposure in patients, including variability in oral drug absorption, distribution, metabolism, and elimination. Additionally, patient characteristics such as age, sex, body weight, renal function, and liver function also contribute to variability in plasma exposure. TDM is the quantification of real-time drug concentrations in blood, and it considers the interindividual variability of pharmacokinetics and enables personalized pharmacotherapy. It is now widely used in many areas of clinical medicine, such as antibiotics, antipsychotic drugs, and immunosuppressants, but its use for anticancer therapies has been limited because of the undefined concentration-effect relationships. Nonetheless, targeted anticancer drugs show a large inter-individual pharmacokinetic (PK) variability and a narrow therapeutic index, which fit many criteria defined as prerequisites for utilizing TDM approaches. TDM has been used to improve the clinical use of chemotherapy agents partly, for example, methotrexate, imatinib, and so on. However, further clinical research evaluating the impact of TDM on overall survival or other pharmacodynamic outcomes needs to be carried out. In the current study, we observed a high inter-individual variability in both C_max_ and C_min_, but the venetoclax peak concentrations were significantly higher in AML patients with AST levels increasing. ALT showed a relevant effect on C_min_ instead of C_max_. AST and ALT levels are predictive indexes of disease and can reflect liver function. Deng et al. and Salem et al. showed that severe hepatic impairment and a stronger inhibitor of CYP3A4, OATP1B3, have an impact on the venetoclax clearance parameter [32,35], so it seems that liver function may be a key factor in inter-individual variability. Besides, intestinal CYP3A4 and P-gp mediated the first-pass metabolism and provided a basis for inter-individual differences in oral absorption while hepatic CYP3A4, OATP1B3 was involved in drug elimination and offered a theory for inter-individual differences in clearance. Thus, the influence of genetic variants on venetoclax concentration needs to be further studied.

## 3. Material and Methods

### 3.1. Chemicals and Reagents

Venetoclax (ABT-199, purity ≥98.5%) was purchased from GLPBIO, [^2^H_7_]-venetoclax (D_7_-ABT-199, purity ≥ 98.5%) was provided by Alsachim, used as an internal standard, and Acetonitrile (ACN) was supplied by Merck&Co., Inc. (Darmstadt, Germany). Ultrapure water was acquired from a Milli-Q^®^ Water Purification System (Millipore-Ibérica, Madrid, Spain). Dimethyl sulfoxide (DMSO) and formic acid were purchased from Sigma-Aldrich (Madrid, Spain). Solvents were all of LC-MS grade. Blank human plasma was obtained from RILD Co. Ltd. (Shanghai, China).

### 3.2. Calibrators and QCs

Stock solutions of venetoclax and [^2^H_7_]-venetoclax were prepared in DMSO at concentrations of 2 mg/mL. The IS working solution consisted of 1μg/mL [^2^H_7_]-venetoclax in ACN. Calibrators were prepared in DMSO at seven concentrations, based on the clinical ranges, and spiked into blank plasma samples at the concentration of 25, 200, 1000, 2000, 4000, 5000, and 8000 ng/mL. The QC samples for method validation at LLOQ (25 ng/mL), low (50 ng/mL), medium (2500 ng/mL), and high (6000 ng/mL) were also prepared by the above method. The amount of solvent added to the matrix, both for calibrators and QCs, did not exceed 2%.

### 3.3. Sample Preparation

Plasma samples were subjected to protein precipitation, and an aliquot of 100 μL sample was prepared in an Eppendorf tube. Subsequently, 300 μL of IS working solution in ACN was added, followed by vertexing for 3 min and centrifugation at 13,000 rpm for 10 min; 100 μL of the supernatant was transferred to HPLC vial and 2 μL was injected onto the UPLC^®^ column.

### 3.4. UPLC-MS/MS Conditions

The UPLC-MS/MS system was comprised of an ACQUITY UPLC I-Class separation module (Waters, Milford, USA) coupled to a Xevo TQ-S micro triple-quadrupole mass spectrometer (Waters, Milford, CT, USA) in positive mode, and Chromatographic separation was conducted on an ACQUITY UPLC BEH C18 column (2.1 × 100 mm, 1.8 μm, Waters) equipped with an in-line filter (0.2 μm), maintained at 35 °C with a gradient elution (0–0.3 min, 5% of B; 0.3–2.0 min, 5–95% of B; 2.0–2.5 min, 95% of B, 2.5–2.6 min, 95%–5% of B, 2.6–4.0 min, 5% of B) at 0.4 mL min^−1^, where mobile phase A and B were 0.1% formic acid in water and acetonitrile (ACN), respectively. The autosampler temperature was set at 4 °C. Under these conditions, venetoclax and [^2^H_7_]-venetoclax (IS) typically eluted at 1.8 and 1.8 min.

Analytes were quantified in multiple-reaction monitoring (MRM) mode at *m*/*z* transitions of 868.5→321.0, 868.5→636.3 for venetoclax and 875.5→321.0, 876.5→636.3 for [^2^H_7_]-venetoclax (IS), respectively. The source temperature was set at 150 °C and the ion spray voltage at 3.0 kV, nitrogen was used as nebulizing (50 L·Hr^−1^) and drying gas (600 L·Hr^−1^) at 500 °C, and the fragmentor voltage (volt, V) and Collision energy (CE, eV) were optimized and set at 70 V, 36 V; 60 V, 40 V for venetoclax and IS, respectively. Data acquisition and processing were controlled by Waters MassLynx software (version 4.2).

### 3.5. Method Validation

The validation of this method was based on FDA guidelines (FDA, Guidance for Industry Bioanalytical Method Validation, 2018.) [36] including calibration curve, precision, accuracy, selectivity, matrix effect, recovery, carry-over, and stability.

#### 3.5.1. Calibration Curve, Selectivity and Specificity

The calibration curve was generated by plotting peak area ratios (analyte/IS) versus the nominal concentration using 7 levels of non-zero standards and a linearly weighed (1/x^2^) least-squares regression model. The error of accuracy and relative standard deviation (RSD, %) should be within 15% of the nominal value for each CAL (*n* = 3) or within 20% for the lower limit of quantitation (LLOQ, *n* = 6) with a signal-to-noise ratio (S/N) ≥ 10 for specificity. The selectivity was confirmed by analyzing spiked blank matrix (commercial blank human plasma) at the LLOQ, and the chromatographic peak response compared with the blank matrix should be less than 20% for the analyte and less than 5% for the IS.

#### 3.5.2. Precision and Accuracy

The intra-day or inter-day precision and accuracy of the method were determined by analyzing the four QC levels (LLOQ: 25 ng/mL, LQC: 50 ng/mL, MQC: 2500 ng/mL and HQC: 6000 ng/mL) in plasma by six replicates on the same day or different three days, respectively. The precision was evaluated as the mean concentration (ng/mL) ± SD and relative standard deviation (%RSD) accuracy was expressed as relative error (%RE), while the value should not exceed 15%, except for LLOQ (RSD < 20%).

#### 3.5.3. Matrix Effect and Recovery

The extraction recovery was determined at three levels (50, 2500, and 6000 ng/mL) by comparing the peak area of MER (samples spiked analyte before blank matrix extraction), MEX (samples spiked analyte after blank matrix extraction) and MEP (reconstitution solvent containing equal concentration).

The equations for evaluation of the extraction recovery (Equation (1)) and matrix effect (Equation (2)) are listed below:

Extraction recovery = MER/MEX × 100%(1)

Matrix effect = MEX/MEP × 100%(2)

The RE obtained for the IS-normalized matrix factor should be less than 15%.

#### 3.5.4. Carry-Over

The blank sample (blank matrix) was injected following the upper limit of quantification (ULOQ, 8000 ng/mL) to understand the carry-over effect. The peak area of the analyte and the IS in the blank sample should not exceed 20% of that obtained for the LLOQ and 5% of the IS, respectively.

#### 3.5.5. Stability

Storage stability was evaluated at low and high QC levels (50, 6000 ng/mL) in three replicates, such as keeping at room temperature for 6 h, −80 °C for 31 days, three freeze/thaw cycles of unextracted samples, and the postpreparative stability of samples in an autosampler at 4 °C for 8 h was also tested. Samples were considered stable if the ratio between original concentration and concentration under different storages was less than 15%.

### 3.6. Application

The method was successfully applied to determine the stable trough plasma concentration (C_min_) and peak plasma concentration (C_max_) of 109 plasma samples from 62 AML patients. Briefly, patients received a daily dose escalation of venetoclax of 100 mg (Day1), 200 mg (Day2), to a final dose of 400 mg (Day3) in 28-day cycles with azacytidine 75 mg/m^2^/day for 7 days. Considering the C_max_ may influenced by fasting and dietary intake, venetoclax was taken after a standardized diet (low-fat meal) within 30 min. No patients were administered with CYP3A4, OATP1B3, P-gp inducers/inhibitors. Blood samples were collected in 5 mL ethylenediaminetetraacetic acid potassium salt (EDTA-K2) tubes on day 10 at steady state, just before the next administration (C_min_) and 6 h after drug administration (C_max_). The blood was centrifuged at 4000 g for 10 min to separate the plasma and then stored in a −80 °C fridge before analysis. The clinical study was approved by the Ethics Committee of first Affiliated Hospital, Zhejiang University School of Medicine (IIT20210153B). All patients provided written informed consent to participate in our study.

### 3.7. Statistical Analysis

Categorical variables were presented as absolute counts. Continuous data were presented as the mean (± standard deviation (SD)) and median (range), and categorical and continuous variables were compared with the Fisher’s exact and a two-tailed Student’s t-test, respectively. Continuous variables were tested for normality using the Kolmogorov–Smirnov test. Correlations were assessed using Pearson’s or Spearman’s coefficient tests. *p*-values < 0.05 were considered statistically significant and a confidence level of 0.95 was used for estimating intervals. All analyses were conducted using SPSS Statistics version 18.0 (IBM Corp., Armonk, NY, USA) and GraphPad Prism version 7.0 (GraphPad Software, La Jolla, CA, USA).

## 4. Conclusions

We described a specific, sensitive, and rapid UPLC-MS/MS method for quantification of venetoclax in human plasma. The method was successfully validated and applied to patient samples with a good performance in selectivity, linearity, precision, accuracy, and stability. In our study, a wide variability in venetoclax concentrations was observed, consistent with the results of previous studies. More importantly, C_max_ and C_min_ of venetoclax showed a positive correlation with AST levels, but ALT had no clinically relevant effect on C_max_. At the same time, C_max_ of venetoclax was significantly higher in the older patients. This makes it necessary to consider TDM could be a useful tool in patients undergoing venetoclax treatment, especially with hepatic impairment.

## Figures and Tables

**Table 1:** Relevant LC–MS/MS characteristics. internal standard, IS.

Compound	Retention Time (t_R_, min)	Transition (*m/z*)		Cone Voltage (V)	Collision Energy (V)
		Quantification	Confirmation		
venetoclax	1.8	868.5→321.0	868.5→636.3	70	36
IS	1.8	875.5→321.0	875.5→636.3	60	40

Internal standard, IS.

**Table 2:** Intra-and inter-day precision and accuracy of venetoclax (mean ± SD, *n* = 6).

Compound	Concentration Spiked (ng/mL)	Intra-Day Concentration Measured (ng/mL)	Accuracy RE (%)	Precision RSD (%)	Inter-Day Concentration Measured (ng/mL)	Accuracy RE (%)	Precision RSD (%)
Venetoclax	25	24.80 ± 0.57	−0.80	2.28	25.12 ± 1.64	0.49	6.51
	50	51.35 ± 3.17	2.70	6.18	48.44 ± 3.54	−3.11	7.30
	2500	2523.77 ± 89.95	0.95	3.56	2419.72 ± 122.66	−3.21	5.07
	6000	5609.22 ± 111.53	−6.51	1.99	5711.31 ± 235.93	−4.81	4.13

**Table 3:** Extraction recovery and matrix effect of venetoclax and IS (mean ± SD, *n* = 6).

Compound	Concentration Spiked (ng/mL)	Recovery (%)		Matrix Effect (%)	
		Mean ± SD	RSD%	Mean ± SD	RSD%
Venetoclax	50	108.36 ± 2.63	2.63	89.77 ± 4.78	5.33
	2500	106.24 ± 1.93	1.82	89.36 ± 3.38	3.78
	6000	106.64 ± 5.73	5.37	91.12 ± 2.02	2.21
IS	1000	104.41 ± 2.76	2.62	90.19 ± 2.34	2.60

**Table 4:** Stability of venetoclax in plasma under different conditions. (mean ± SD, *n* = 3).

Spiked (ng/mL)	Condition	Found (ng/mL, Mean ± SD)	Precision RSD (%)	Accuracy RE (%)
50	Room temperature for 6 h	45.67 ± 1.12	2.4	−8.66
	−80 °C for 31 days	49.57 ± 1.15	2.3	−0.87
	Three freeze-thaw cycles	48.77 ± 1.53	3.1	−5.38
	Autosampler in 4 °C for 8 h	46.70 ± 2.13	4.6	−6.60
6000	Room temperature for 6 h	6045.47 ± 152.82	2.5	0.76
	−80 °C for 31 days	5618.17 ± 56.22	1.0	−6.36
	Three freeze-thaw cycles	5676.83 ± 71.15	1.3	−2.5
	Autosampler in 4 °C for 8 h	5800.63 ± 168.44	2.9	−3.32

**Table 5:** Patients’ demographic data and clinical characteristics. (*n* = 62).

Characteristic	Mean (±SD)	Median (Range)
Age (year)	58.9 ± 14.3	62 (18.0–90.0)
Height (cm)	165.5 ± 5.5	165.0 (153.0–177.0)
Weight (kg)	63.44 ± 10.48	62.25 (43.5–92.0)
BMI (kg/m^2^)	23.1 ± 3.3	23.0 (16.9–31.46)
Gender (Female/Male)	26/36	
ALB (g/L)	40.1 ± 5.65	40.85 (25.7–51)
ALT (U/L)	15.08 ± 8.65	14 (3–54)
AST (U/L)	20.45 ± 10.66	17 (4–62)
BUN (mmol/L)	5.83 ± 2.61	5.41 (1.94–18.70)
CREA (µmol/L)	77.4 ± 30.6	69.5 (42.0–224.0)
LDH (U/L)	358 ± 325.1	234.5 (54–2066)
WBC (10^9/L)	9.37 ± 14.34	3.94 (0.77–83.59)
NEUT (10^9/L)	2.60 ± 5.21	1.485 (0.06–31.29)
HGB (g/L)	86.4 ± 24.85	83 (45–137)
PLT (10^9/L)	85.18 ± 151.9	33.5 (3–1097)
C_max_ (ng/mL, *n* = 61)	2966.0 ± 1595.0	2408.0 (515.4–7189.0)
C_min_ (ng/mL, *n* = 48).	1018.0 ± 729.4	836.6 (74.2–3257.0)

ALB, albumin; ALT, alanine aminotransferase; AST, aspartate transaminase; BUN, blood urea; CREA, creatinine; LDH, lactate dehydrogenase; WBC, white blood cell count; NEUT, neutrophil; HGB, hemoglobin; PLT, platelet.

## Data Availability

All data included in this study are available upon request by contact with the corresponding author.

## Supplementary Materials

The following are available online, Figure S1: The comparison of AST (A) and ALT (B) levels before and after treatment, Table S1: Summary of analytical performance for quantification of Venetoclax.

## Sample Availability

Samples of the compounds are not available from the authors.
