# Supplementary material for: Quantification of Venetoclax for Therapeutic Drug Monitoring in Chinese Acute Myeloid Leukemia Patients by a Validated UPLC-MS/MS Method
Source: Molecules. 2022 Feb 28;27(5):1607. doi: 10.3390/molecules27051607 (PMC8911561; doi:10.3390/molecules27051607)
Supplement: Supplementary file 1 [file molecules-27-01607-s001.zip › molecules-1545656-supplementary.pdf]

Table S1. Summary of analytical performance for quantification of Venetoclax

| Reference | Column | Mobile phase | Internal standard | Run time | Sample preparartion | Instrument | linearity | Method validation | Matrix |
|---|---|---|---|---|---|---|---|---|---|
| This study | ACQUITY UPLC BEH C18 column (2.1×100 mm,1.8 μm) | 0.1% formic acid in water and acetonitrile | [$^2H_7$]-Venetoclax | 4 min | deproteinization | LC-MS/MS | 25-8000 ng/ml | Yes | Human plsama |
| Choo, et al., 2014 | Phenomenex Kinetex C18 column (50× 2.1 mm, 2.6-mm) | 2 mM ammonium acetate in water and acetonitrile with 0.1% formic acid | NA | 1.6min | deproteinization | LC-MS/MS | 0.005-10 mM | NA | Dog plasma |
| Liu, et al., 2017 | Phenomenex Synergi Polar-RP, 4.6 ×250 mm, 5 mm) | 25 mM ammonium formate aqueous solution (pH 3.5) and acetonitrile | [$^2H_8$]-venetoclax | >60min | solid phase extraction, liquid-liquid extraction | Liquid Scintillation Counting ， HPLC | 82.5 ng/g- 87.2 ng/g- 377 ng/g- | NA | Human Fecal, urine and plasma |
| Eisenmann, et al., 2014 | C18 AQUASIL guard cartridge (2.1 × 10 mm, 3μm) | 0.1% formic acid in water and 0.1% formic acid in ACN-MeOH (50:50, v:v) | [$^2H_7$]-Venetoclax | 4.6 min | liquid-liquid extraction | LC-MS/MS | 5–1000 ng/mL | Yes | Mouse plasma |
| Reddy, et al., 2014 | Phenomenex Kinetex C18 (150 × 2.1 mm, 2.6 μm) | 10mM ammonium formate and 0.1% formic acid with acetonitrile | ribociclib | 6 min | upper layer of deproteinization diluted with water | LC-MS/MS | 5-500 ng/mL | Yes | Rat plasma |

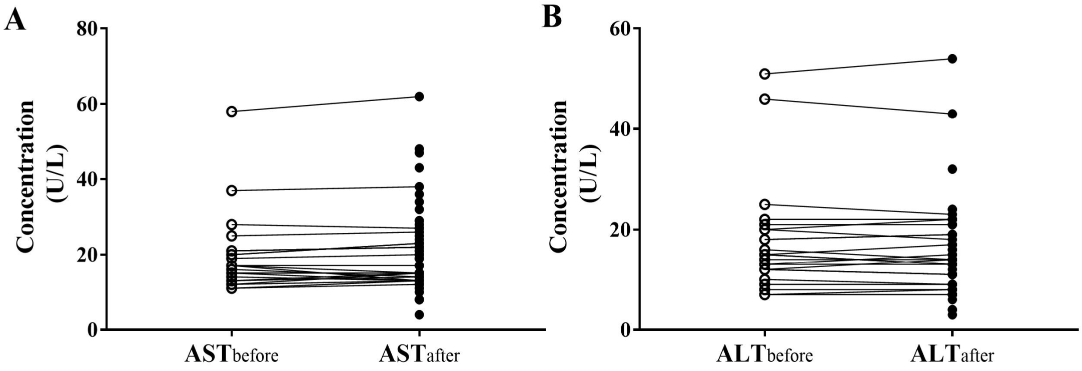


Figure S1. The comparison of AST (A) and ALT (B) levels before and after treatment.
